# Paralysis and Convulsions as Effects of Diseases of the Base of the Brain

**Published:** 1878-03

**Authors:** 


### Original Lectures.

### PARALYSIS AND CONVULSIONS AS EFFECTS OF DISEASES OF THE BASE OF THE BRAIN.

DELIVERED IN CHICAGO FEBRUARY 21ST, 22D AND 23D, 1878.

BY DR. BROWN-SÉQUARD.

*(Reported for the Journal and Examiner.)*

### FIRST LECTURE.

GENTLEMEN: One of the propositions I have in view in this course of lectures, is to try to open a new view of the therapeutics of this subject. Yet, although this is one of my purposes, I will say that only a part of the three lectures will be given to that peculiar object of this course. Not that there would not be a great deal to be said on that point. On the contrary, I delivered once sixteen lectures on that very small part of the lecture to be delivered here. But it is more important precisely to bring you to understand what those individual views about treatment can be and are now. It is more important perhaps to dwell at length, as I need not say, upon the nature of convulsions as related to disease in the base of the brain. It is more important to do so because of the very few who will follow out the facts which I have to introduce on these two subjects of paralysis and convulsions. These views will give the key and render you able by yourselves, without my assistance, to follow up in your own practice the general principles of treatment which I will mention in the third lecture.

I now come at once to what is to be the subject of the two lectures. And to begin with, there is, as you well know, a pretty general acceptation at present of the views that have resulted from the experiments recently made by Hitzig, Flint, and other physiologists, as regards the existence of certain centers in the convolutions of the brain. I do not intend to give much time to that part of the subject; but it is quite essential that I should say that taking these new views together with the old ones, it is now admitted, as you well know, that paralysis in cases of disease of the base of the brain, as well as in diseases elsewhere, occurs from the destruction of tissue in parts which are employed here as centers for volition or conductors of volition. Paralysis, therefore, is considered as the direct effect of the disease existing in the part of the brain as found in the autopsy. As regards convulsions in cases of brain disease, they are construed now by a great many physicians as dependent upon putting in play the nerve cells where the disease is, especially when there is disease of the surface of the brain, when there is disease in the motor zone of the brain and behind the fissure of Rolando.

Convulsions are thus considered as being the manifestations of the properties and functions existing in those parts. I will have to show, by-and-by, that this last view, as well as those relating to paralysis are absolutely false. For the present, to continue this exposition of what is admitted, I will say that since the time that paralysis has been observed to come usually in the side of the body which is opposite to that of the seat of the lesion in the brain, it has been considered that one side of the brain is the mover of the opposite side of the body. This I will contradict absolutely; and perhaps some arguments which I will bring forward will convince you that the view must be given up.

It is admitted also that decussation, which we know to exist at the foot of the anterior pyramid of the *medulla oblongata*, contains most of the voluntary motor nerve fibers, and after the discovery of the anterior pyramids the explanation came of what was known before this, that paralysis in the left side of the body is caused by disease in the right side of the brain. A series of facts, which I am glad to have published, which I collected right and left (a very few seen by myself also), seem to corroborate these views. I will first mention a few of these facts so as to show you how the question stood before I began to criticise not only the views generally admitted, but the views I had helped to establish.

If you take the case of disease of one of the *cerebra,* it is usual, as you well know, (if it is the right *cerebrum*), that the left side of the body will be paralyzed while the right will escape altogether. Such facts seem to show that if decussation exists, as is supposed, it is certainly below that part. Conceive that decussation takes place in the *crura cerebri*, that this decussation contains fibers which are employed in voluntary movements, you could not say, as is observed almost always in these cases, that the paralysis is limited to the side opposite to that where the disease is in the brain. It is easy to understand this point. [Exhibiting a drawing on the blackboard.] It is necessary to destroy the fibers belonging to the two sides of the brain. Although the lesion is on the left side, it destroys the fibers coming from the left side going into the right side, and destroys the fibers coming from the right side and going into the left side. Therefore, if the decussation of the voluntary motor conductors is there, you would not see, as is seen, this complete paralysis of the opposite side, and no paralysis at all on the corresponding side.

The same argument can be applied as regards the *pons varolii*. Many anatomists have selected the decussation of the fibers of the left *pons varolii*, and you know certainly that a good many physiologists admit that the fibers that decussate there form a part of the voluntary motor apparatus. But the same argument that I used a moment ago would show that we must reject the view that is admitted. [Drawing an illustration on the blackboard.] I will make this fiber come from the left side, and another from the right side. If they decussate it necessarily destroys the fibers belonging to the two sides of the brain. There ought to be paralysis of two sides, although the lesion is on only one side. I collected, in two papers published long ago, something like forty or fifty-five cases of disease of half of the *pons varolii*, showing that paralysis was located, in all cases, only on the opposite side; so that these facts seem to be clearly in opposition to the view that there is decussation at that place. The conclusion which I drew from these facts was that the decussation takes place only in the *medulla oblongata*. The arguments seem extremely powerful, but, as you will see in a moment, other facts have come since, which corroborate the facts I have mentioned. There is no doubt whatever that, in cases of lesion above the anterior pyramids which produces a paralysis on the opposite side only, and not at all on the side corresponding—that this is a very strong argument in favor of the view that if decussation is necessary, if the voluntary motor conductors decussate somewhere in the base of the brain, that decussation occurs below the *pons varolii*, or in the *medulla oblongata*. This is certainly easily seen. It was admitted for a long time, in our century, indeed, or about twenty years ago, that if paralysis comes on the opposite side of the disease in the base of the brain, it exists on the opposite side because decussation takes place only in the anterior pyramid and produces paralysis on the opposite side, though there are exceptions. I will try to prove (and in very few words and few arguments, because the time is so short), that the views I have to propose are absolutely different from these.

In the first place I will try to show that one half the brain is perfectly sufficient for the movement of the two sides of the body.

In the second place I will try to show that a few fibres establishing connection between one side of the brain and the spinal cord, are perfectly sufficient for most of the movements of the brain.

In the third place, contrary to what is now admitted, as regards instances of certain so-called psycho-motor centers, I will try to show that an agglomeration or cluster of cells located in the immediate part of the brain, the cells employed in moving for instance the arm, and serving therefore for a center, or the leg or any other important mass of muscles—I will try to show that such an agglomeration of cells does not exist anywhere, and that the cells which are employed in moving the arm for instance, are scattered all over the brain, so that destruction may take place in any part of the brain without any paralysis of the arm ; all these cells, however, serving to move the one limb, or any other part, being connected by fibres so as to establish a normal state of communication between all those which have the same function.

I will show, or try to show, something more, which is that it is not any mere nerve current going from the cells of the brain which is employed in moving muscles—not merely a nerve current going from those cells straight to the muscles which are to be moved, when movement is performed. I will try to show that it is absolutely essential to admit that through a very few of these wires, which we call nerve fibers, a real telegraphic message is sent to cells which execute the movement according to the order conceived in the message, and therefore that voluntary action is entirely different from what it has been imagined to be.

As regards paralysis, I will say that it does not appear, as I have admitted for many years, to result from a destruction of the parts endowed with the function that disappears. Paralysis comes from something quite different. If you take, for instance, a case of disease in one of the *crura cerebri* which I have named, it is not because conductors employed in voluntary efforts are destroyed by the disease which we find there; it is not for that reason that paralysis appears. It is because irritation starts from the place where the disease is—irritation propagated through cells to a distance, so as to act on these cells in the same way that we know the *par vagum pneumogastric* acts upon the cells that move the heart when the *par vagum* is galvanized. In other words, paralysis from brain disease, either when the disease is in the base or elsewhere, I will show to depend upon an influence exerted by the disease on parts at a distance, so as to stop the activity of the cells in it that produce the functions which disappear. It is not where the disease is that we find the real paralysis exists. The paralysis is distant from where the disease is located.

As regards convulsions, the very same thing exists. I will try to show that convulsions appear to result from spasms of irritation, beginning at the part where the disease is, and acting on cells at a distance so as to put them in play. In other words, as regards those two kinds of phenomena, it is association of activity of muscles, or putting the muscles into great activity. These two states of symptoms depend on something quite similar. Irritation in the two cases, beginning with the disease, is propagated to cells at a distance so as to do one of two things, that is, stop the activity of the cells and produce paralysis, or to put these cells in play, giving rise to a discharge of nerve force producing convulsions.

Now, that you have face to face the two theories, the old and the new, I will proceed to the demonstrations, or to attempt the demonstrations.

In the first place, one of the facts on which it has been supposed that a decussation takes place in the *medulla oblongata* (which decussation comprises the mass of nerve fibres employed in the voluntary movements)—a fact which I have not mentioned, and which seems to be extremely favorable to the views admitted generally, is that in cases of brain disease, especially in cases in which the middle lobe is affected, there is a secondary degeneration which starts from the place of the disease, thus coming from the *crura cerebri*, reaching the *pons varolii* on one side, and then one of the anterior pyramids, and then changing its direction passing into the posterior part of the lateral parts of the spinal cord. For a time, when this was discovered by Bichât, it was thought the arguments were in favor of the voluntary motors decussating in the anterior pyramids. As there is a notion that when the cells from which the motor fibres originate are destroyed, that the motor fibres will degenerate, it was supposed that the motor bundles in the *crura cerebri* had their cells of origin in the place of the disease, and that their degeneration was similar to what we find when a motor nerve is divided, as Waller had established. But you will see what difficulties there are.

In the first place (if we look upon the anterior pyramids as being what I with others supposed for a long time the channels of orders of the will to the muscles) we are led into strange contradiction with experimental facts and clinical facts relating to the spinal cord. The anterior pyramids, as is well demonstrated, after passing into the lateral parts of the spinal cord do not pass into the anterior columns. Both physiological and clinical facts, and many more clinical facts than even experimentation, have shown that the lateral columns are not the channels through which the voluntary motor fibres pass to the muscles. I know that recently Ludwig and a pupil of his have tried to show that the lateral columns contain most of the fibres; but it is difficult to admit that there are no mistakes in his experiments, as all other physiologists have ascertained that section of the lateral column is invariably followed by paralysis of the other side of the body. We have performed the experiment many hundred times, and never saw the least trace of paralysis. But it is quite certain that in a part of these cases all the lateral columns had been divided. But, I repeat, clinical facts are more peremptory. There are many cases in which the lateral columns have been destroyed, either by a tumor or by cutting, when there has been no paralysis at all. So that it is quite certain that the fibres of the lateral columns of the anterior pyramids cannot be the parts through which the orders of the will pass to the muscles.

As regards the secondary degeneration, the argument is strong against the view that the pyramids are channels or instruments of the orders of the will to the muscles. As we see, the secondary degeneration takes place only in the posterior part of the lateral columns. It is not the lateral column that degenerates; it is only those that are near the origin of the posterior nerves. So that we can look upon the secondary degeneration as being degeneration of the voluntary motor conductors. And the argument is very strong in this case that if paralysis occurs together with secondary degeneration, it is not the fibers which are employed in moving the muscles; it is not the fibers which are paralyzed that are so degenerated. There must be other fibers which do not seem to have degenerated at all, which have lost their function. Indeed these two series of facts relating to the experiments and clinical facts, relating to the lateral columns of the cord, all those also relating to secondary degeneration clearly show that the mass of the nerve fibers which we find in the *pons varolii*, which we find at the junction of the pyramids and in the *cura cerebri* are not the parts containing the voluntary motor conductors.

But there are more clear and positive facts than these. At one time I, with a number of other physiologists, found that in rare instances in which it had been possible to divide a good part of one of the anterior pyramids and sometimes of both of them—in those very rare experiments of a division of a part of these pyramids, we found that paralysis was produced by the operation. Magendie has the merit of having succeeded more fully than other physiologists, and indeed, of those of my time as well as of preceding generations, of having performed the experiments in a more complete way than others. He found that division of one anterior pyramid does not produce paralysis, and division of the the two pyramids does not produce paralysis. Since that time better modes of experiment have been found, and Schiff and I have made the experiment and others have ascertained that the anterior pyramid on one side can be cut without marked paralysis, and that both pyramids can be cut without marked paralysis.

If you put this experimental fact in the presence of some clinical facts which are strong in their significance, disease of the anterior pyramids does not always produce paralysis, and I have known of their lesion 22 times without paralysis. In one case a woman who worked in the hospital up to the day that preceded her death had considerable degeneration of the anterior pyramids, and probably a few fibers remained normal in those parts.

In another case noted by an observer whom I name more willingly than others owing to his accuracy, there was disease of the spinal cord and paralysis of the lower limbs, but the upper limbs were free, and the two anterior pyramids were destroyed. Dr. Bichât, who is also a very able man, has had cases of that kind, and a good many such have been observed. But in other cases another kind of alteration takes place, that which comes from a partial abscess located there, and in these cases also no paralysis occurs, or at least no marked paralysis has been observed so that both experimental physiology or rather experiments on animals, and observed clinical facts establish that the anterior pyramids can be divided or destroyed by disease without any marked paralysis. What becomes, therefore, of the view that they are the only channels that serve to establish communication between the will and the muscles ?

Now, if you put this series of facts in the presence of the other series of facts, that the location of the disease in one of the *crura cerebri* or in the *pons varolii*, has produced paralysis only on the opposite side, and if you remember the conclusion I derived from these facts, and the conclusion which flows fully from these facts, you will see that it is impossible to admit that the voluntary motors decussate there. If you scan the facts closely, you will find that there is no place in the base of the brain where it can be demonstrated that the voluntary motors decussate.

And now you come from this demonstration to the conclusion that if you admit decussation, you must place it elsewhere than in the base of the brain. And I will ask where? Certainly it is not above the *crura cerebri.* For, if it is there, the same arguments would be applicable to it; and certainly not in the spinal cord, where we know there is decussation all along and crossing the fibers going from the anterior columns in front of the central canal of that organ. It is not there that we are to place decussation, and one reason is sufficient. It is that when we find disease located in one half of the spinal cord, it produces paralysis of movement only in the corresponding side, so that there is no possibility of admitting that decussation which we see in the cord as a physiological decussation of the voluntary motor conductors.

We are now, as you may observe, in the presence of a complete annihilation of the views that are admitted as regards the general form of paralysis in brain disease. We are in the presence of facts which, on the one hand, oppose the generally received view that where there is disease in the left side of the brain, paralysis is in the right side of the body, and *vice versa* for the other side of the brain. We are in the presence of that fact which certainly seems to show that there must be a decussation of conductors between the left side and the muscles on the right side of the body, and generally with the fibers from the opposite side. And still we find, by the arguments I have presented, that there cannot be such decussation. What, then, is the explanation of paralysis? We must reject necessarily the old explanation, and coming now to another series of arguments, and taking only for a moment (as it is somewhat out of my subject), the voluntary motor apparatus at the psycho-motor centers, what do we find as regards disease there? (I do not speak of experiments that I could show—there are many of them.) Let us consider what is more interesting, only the clinical facts. There is no question that disease in those pretended psycho-motor centers will produce paralysis frequently. I would not say very frequently, but certainly frequently. And there is no doubt also that out of a hundred cases of paralysis produced by disease located in that part, you will find more than eighty cases. You will find that in most cases a lesion, limited to a small part of that zone, causes paralysis. Although it is only a small part of that zone, you may find paralysis of the two limbs on the opposite side. How could it be so, as most of the zone, which is called psycho-motor, remains perfectly healthy? How is it that the destruction of a part of the zone produces complete paralysis of the opposite side? In many of these hundred cases you will find it is paralysis of the arm. A paralysis of the leg or arm will come, in perhaps one case out of a hundred, and when it comes it will give light to the theory, because it will be placing out of tone, considered as a zone, the psycho-motor organ and the leg. Now, the discrepancies between the meanings of the effects and the place of beginning of the paralysis, and the number of parts becoming paralyzed—the discrepancies are such that it is impossible to admit these views.

But take paralysis of the arm. It has been proposed—and I must say that I am sorry that such view has been put forward, because it may mislead a great many surgeons, and perhaps may prove absolutely wrong—on the mere presence of paralysis of the arm (in case of, for instance, a blow upon the head) to apply the trephine in accordance with the ordinary rules at the place considered as the part of the brain which moves the arm. But if you know how many facts there are which show that paralysis of the arm may come from disease in any part of the brain—if you know that a blow on the head will almost always produce paralysis—if you know that, what then, as regards the application of the trephine? How can you be entitled to apply it at the place supposed to be the psycho-motor center of the arm? As regards a great many arguments against these views I will only add this: that we may see paralysis occurring in the arm or the leg, or in both, when the disease is at the surface of the brain, or in the front of the psycho-motor center, or far from it in the posterior part of the brain, or the anterior part, so that indeed those views about motor zones are in complete contradiction with what clinical observation teaches.

Another series of arguments which I have no time to mention, is that those two sets of psycho-motor organs can be destroyed without any paralysis.

I pass now to other parts of my demonstration. If you examine what takes place in cases of disease of a certain part of the brain which I have been led to call the special corner of the brain—a corner which forms a kind of angle limited by the *cerebra*, the *pons varolii*, and the *medulla oblongata*—in that special corner, when disease exists, you find it may show itself elsewhere more decisively and more strikingly. In that peculiar part of the brain we find more centers of disease than elsewhere and paralysis occurring on the corresponding side of the body. My experience is limited as regards the dissection of dead bodies; but I have seen three such cases in which the autopsy was made, and I have been able to diagnose during life, cases of this disease which have been followed by autopsy. The case was perfectly clear. There was no difficulty in coming to the conclusion I arrived at, and therefore I can say I made the diagnosis, and the autopsy approved it. I shall have to say in the third lecture what were the results of the diagnosis in that case as well as other cases. For the present, what I wish to say is, that in that special part of the base of the brain, a lesion produces paralysis on the same side more frequently than on the opposite side. Lesion there also produces paralysis of the two lower limbs, sometimes paralysis of the two upper limbs; although (I repeat) it is located in one side only of the base of the brain. It may (and it is so in a great many cases, and perhaps more frequently than in other cases) produce no paralysis at all. So that here is the same lesion, pretty much the same in nature as in most cases of the disease, temporary degeneration of the *dura mater* and petrous bone; in most of these cases having destroyed pretty much the same parts in the base of the brain; but while it produces no paralysis in some cases, in other cases it produces paralysis of the same side, in still other cases paralysis on the opposite side, in other cases paralysis of the two lower limbs, and in others paralysis of the two upper limbs. How can you reconcile such facts with the view that the paralysis arises from the destruction of the tissues, where we find the disease after death? Certainly this is impossible. You cannot admit that in one individual the fibers are destroyed on the same side, and in another the fibers that go to the muscles on the opposite side, while in another they are the fibers extending to the lower limbs, and in another the fibers that extend to the upper limbs, and in others the fibers that go nowhere. Indeed, it is absolutely impossible to imagine the variety of function that would be adequate to produce the variety in morbid manifestations that we see in these cases. Hence, therefore, at least for these cases, we must decline to accept, that paralysis there appears from a destruction of the fibers employed as conductors between the will and the muscles.

There is, as regards convulsions in connection with the base of the brain (and especially that corner, that angle) pretty much the same variety that I have alleged to exist for paralysis. Convulsions may appear anywhere and everywhere at the same time, so that the greatest variety of effects, both as regards convulsions or paralysis, can come from disease located in that place. It may be supposed that in some of these cases, at least, you could not, by any supposition that I can foresee, explain all the effects; but it may be admitted that fibers exist there which do not decussate, but which come directly from the brain to the muscles. Indeed this supposition has been made. Some physiologists have admitted the theory that voluntary motor fibers when they decussate, do so in the base of the brain in many parts. The part that does not decussate exists there in that special corner which I have mentioned. This would seem to be in harmony with certain facts. Thus, the fibers on the lateral column of the *medulla oblongata* go into the anterior columns of the spinal cord, which we can divide in animals and produce paralysis, and which very rarely indeed are destroyed in man with considerable paralysis in the four limbs of the body if the lesion is high up. Here are these facts, which are clear and evident, although perhaps not so plain as is believed. Here are these facts, still you find on attention to these anterior columns in the *medulla oblongata*, where they form the lateral columns, that they can be divided without any marked paralysis, and in man if these lateral columns be divided by disease or cut, there will be, after such lesion, the variety of effects I have mentioned or no effect at all. How can it be? It is owing to the fact that the anterior columns are parts of the voluntary motor apparatus, that paralysis appears when they are destroyed or divided, that the degeneration in the *medulla oblongata* differs from them so radically. You see the kind of contradiction which renders almost impossible the view that paralysis (even when the disease is in the spinal cord) depends upon the destruction of the conductors. What, then, is the cause of paralysis? A good many facts, which I will mention at length in the last lecture, throw great light on this subject. I will mention only one now, that you may have one fact showing that paralysis can appear from other causes than the destruction of tissue. Perhaps you are aware (certainly I dare say most of you are aware) that if the lateral half of the spinal cord be cut, there is paralysis of movement on the corresponding side below the needle and anæsthesia on the opposite side. There are other features, but I pass them aside. If we prick with the finest needle certain parts of the posterior columns on one side of the dorsal lumbar enlargement, we find sometimes that the animal exhibits identically all the features that we find when the lateral half of the cord has been divided. There is mere irritation of a minute part of the fibers, and that irritation produces identically the same effects that are produced by section of the lateral half of the cord. If such effects can come from such slight causes, you can easily understand that causes more powerful can produce similar effects. Indeed, it is quite certain that a great many facts point out that paralysis, in cases of brain disease, has no relation whatever to the extent of the disease—no essential relation with the seat of the disease. It has no relation as regards its degree, extent or duration. There is no law that can be fixed establishing the relations between paralysis and the disease in the brain.

There are many other points which I might mention in this lecture, but, as time presses, I will hasten to say a few words on a point relating to the appearance of paralysis when disease exists in the *pons varolii.* There is much argument, as you well know, as to whether the right half of the brain contains the voluntary motor conductors for the left side of the body, and *vice versa* whether the left side, the voluntary motors for the right side. Well, it is not so by any means. What I have said of that special corner can be said in a measure of the *pons varolii*, and I will say at once, that disease if located there in one half of the cord, can produce paralysis wherever it exists, whether in the anterior, lateral, or posterior part. It is quite certain that if this is so, when the disease is in the posterior part of the organ, paralysis does not appear, owing to destruction of the conductors, as no physician or anatomist has placed the conductors there. Therefore, there are cases clearly in opposition to the views admitted.

But now, if we examine, what takes place when the disease is in the front part of the *pons varolii*, we find the greatest variety. The disease limited to a part of that organ may produce complete paralysis on the opposite side; on the contrary, the disease may destroy the whole anterior part of the *pons* and yet produce very little paralysis, and in a few cases has produced hardly any paralysis. On the other hand, lesion there, just as in the spinal cord, can produce paralysis on the same side. There are not many such cases; but three are the clearest; one by Dr. Stanley, of London, a very able observer. In that case half of the *pons varolii* was destroyed, and paralysis occurred on the opposite side. The variety as regards extent, duration and degree, therefore can exist without any corresponding variety in the disease of the *pons varolii.*

Another argument relating to convulsions, comes very strongly also from cases of diseases in that part, because the disease there can be diagnosed in most cases, whatever the seat of the paralysis. But convulsions can help the diagnosis. Convulsions may appear any where, and the greatest variety may exist as to their seat. But many cases of brain disease may exist without any convulsions at all.

As you may have seen so far, there are parts in the base of the brain which will give rise to very much the same lesion, but a great variety of effects as regards both convulsions and paralysis.

In the next lecture I will take this part up again, and in the third lecture I intend to give my conclusions relating to the therapeutics of brain disease.

---

Dr. Fuller, of Montreal, has trephined portions of skull of an idiot two years old. The mental improvement was marked. The good doctor, if he is not too busy in his own country, has a vast field of usefulness open for him in this. Cannot he be induced to come over?

